# Ovarian cancer: Pathogenesis and current recommendations for prophylactic surgery

**DOI:** 10.4274/jtgga.galenos.2018.2018.0119

**Published:** 2019-02-26

**Authors:** I Nyoman Gede Budiana, Michelle Angelina, Tjokorda Gede Astawa Pemayun

**Affiliations:** 1 Department of Obstetrics and Gynecology, Udayana University Sanglah General Hospital Faculty of Medicine, Bali, Indonesia

**Keywords:** Ovarian cancer, primary prevention, salpingectomy, salpingo-oophorectomy

## Abstract

Ovarian cancer is one of the most common gynecologic cancers, and one of the leading causes of cancer-associated female mortality in the world. Currently, no widely accepted pathogenesis is available, which may explain the entire disease. Early detection and primary prevention of ovarian cancer are difficult, mostly due to its heterogeneous nature. Risk factor modification based on epidemiologic data has not significantly reduced the incidence of ovarian cancer. Currently, prophylactic surgical methods have been proposed as the most effective preventive measures for both the high-risk or low-risk populations. Understanding the existing pathogenesis theories and the surgical options available may alter physician’s perspectives and facilitate better decision making.

## Introduction

Ovarian cancer is one of the most prevalent gynecologic cancers in Indonesia, associated with high mortality rates (1,2). Difficulties in early detection contribute to the high mortality rate. Most patients (>75%) are diagnosed at a more advanced stage (stage III/IV), with a 5-year survival rate less than 30% (3,4).

Until recently, various preventive and early detection methods for ovarian cancer have not achieved satisfying results, which is partly due to its heterogeneous nature (5,6). Previously, primary ovarian cancer prevention was concerned with risk factor modification and encouraging protective factors, according to epidemiologic data, such as the use of oral contraception. Unfortunately, these modifications have not significantly reduced the incidence of ovarian cancer (7,8). 

Currently, an alternative method has been proposed for ovarian cancer prevention. Prophylactic salpingectomy has been considered the most effective ovarian cancer prevention (9). In 2010, The British Columbia Ovarian Cancer Research Group (OVCARE) started the campaign for prophylactic salpingectomy implementation in hysterectomy and female sterilization. This approach is supposed to reduce the incidence of ovarian cancer as much as 20-40% in the next 20 years (8). This method is not popular yet in Indonesia. Through this review, we aim to provide a new insight and detailed overview of the role of salpingectomy for ovarian cancer prevention.

### Epidemiology of Ovarian Cancer

Ovarian cancer ranks as the fifth leading cause of malignancy-associated mortality in females (10,11). In 2008, an estimated 225,500 women were diagnosed as having ovarian cancer worldwide, and in 2012 it was estimated that there were 238,700 new cases, and 151,900 women died of ovarian cancer (12). In general, ovarian cancer is more common in developed countries than developing countries with the highest incidence in Northern Europe (13.3 per 100,000 per year) and the lowest incidence in North Africa (2.6 per 100,000 per year). In Asia, the estimated incidence of ovarian cancer in China is 3.2 per 100,000 per year (12). In Indonesia, there are no national data on the incidence of ovarian cancer, but in 2002 it was estimated that 829 new cases were diagnosed (2). The incidence of ovarian cancer increases with age, with a peak incidence at the age of 50-60 years (3,4).

### Risk Factors for Ovarian Cancer

Studies showed that women with early menarche (age <12 years) and late menopause (age >50 years) were at higher risk for ovarian cancer due to a higher number of ovulatory cycles. Women with early menarche and late menopause are at a risk of 1.1 to 1.5 times and 1.4-4.6 times higher for ovarian cancer, respectively. Conversely, breastfeeding, pregnancy, and the use of oral contraceptive pills, which suppress ovulation, are protective factors for ovarian cancer (5,13). Epidemiologic studies have shown a link between the incidence of endometriosis and ovarian cancer through an uncertain mechanism (14).

One of the most important risk factors for ovarian cancer is a genetic factor. Genetic predisposition is found in 10-15% of cases of ovarian cancer. BRCA1 and BRCA2 gene mutations are associated with ovarian and breast cancer (5). BRCA1 and BRCA2 were first discovered in 1994 and 1995, and to date are the genes that have the strongest influence with ovarian cancer incidence (15). BRCA1 is an oncosuppressor gene on chromosome 17q21, and BRCA2 is located on chromosome 13q (5). Deletion or insertion of these genes causes the codon to stop prematurely and the protein produced becomes shorter. The genes also play a role in the chromatin remodeling process, thus their mutation causes uncontrolled cell growth. Mutations of BRCA1 and BRCA2 are associated with the risk of ovarian cancer at 50% and 20%, respectively (15).

### Pathogenesis of Ovarian Cancer

To date, no widely accepted pathogenesis of ovarian cancer has been described. One of the biggest problems in uncovering the pathogenesis of ovarian cancer is the heterogeneous nature of ovarian cancer, comprising various histologic types with different behaviors and characteristics (16). Although 40% of ovarian tumors are nonepithelial types, only 10% of ovarian cancers are nonepithelial (17,18).

### Incessant Ovulation Theory

Initially, all ovarian cancers were thought to originate from the epithelium of the ovarian cell surface. During ovulation, these surface epithelial cells experience physical trauma, which is repaired immediately. During a woman's life cycle, ovulation occurs repeatedly, which causes repetitive trauma to the epithelium, ultimately causing cellular DNA damage. Epithelial cells that have undergone DNA damage are very susceptible to change, which facilitates invagination to the cortical stroma. This invagination eventually becomes trapped and forms a sphere of epithelial cells in the stroma called cortical inclusion cysts. While inside the ovary, the epithelial cells are exposed to ovarian hormones that stimulate cell proliferation, which in turn transforms into cancer cells (3,7).

This theory is consistent with epidemiologic data where the number of ovulatory cycles is associated with the risk of ovarian cancer. The weakness of this theory is that it cannot explain the pathogenesis of various histologic types of ovarian cancer and prognostic differences (19). Histologically, the ovarian surface epithelium (mesothelium) has no similarity to serous, endometrioid, mucinous, clear cells or transitional cells (6). In addition, this theory also contradicts the fact that in patients with polycystic ovary syndrome who experience a decrease in the ovulation cycle, the risk of developing ovarian cancer is higher (3,7).

### Fallopian Tube Theory

Previously, most researchers believed that ovarian cancer originated from the ovary itself. Thus, only a few tried to look for ovarian cancer precursor lesions elsewhere (6). It was reported that epithelial dysplasia was found at a high incidence in the Fallopian tubes (50%) of women with BRCA1/2 gene mutations undergoing prophylactic salpingo-oophorectomy. This epithelial dysplasia resembled high-grade serous ovarian carcinoma, which they called tubal intraepithelial carcinoma (TIC). Other studies also found similar histology characteristics of ovarian cancer and high-grade serous peritoneal cancer, regardless of BRCA status. Studies that examined the contralateral ovary of patients with ovarian cancer showed either normal histology or morphologic changes that did not resemble high-grade serous neoplasm characteristics (3,6). Based on these studies, it can be concluded that the fallopian tube would likely be the location of the ovarian cancer precursor lesions, which eventually spread to the adjacent ovary.

TP53 gene mutation is also obtained in TIC. In normal fallopian tubes, immunohistochemical examinations revealed that TP53 expressions in the secretory cells were identical to TP53 mutations in serous ovarian cancer. Nevertheless, not all TP53 mutations become cancerous. TP53 expression is thought to be a response that shows DNA damage in tubal epithelial cells due to exposure to cytokines and oxidants. About 50% of TP53 mutations eventually become cancerous (7,16).       

Almost all TICs (70-90%) are found in the fimbria region, which is the distal part of the fallopian tube. Although initially controversial, this theory began to be accepted by experts. Fimbriae located very close to the ovary are exposed to the same environmental stressors as the ovary. In addition, fimbriae are also rich in blood vessels that facilitate metastasis to the ovaries through the bloodstream (6).

### Two-Pathways Theory

**Figure 1:**
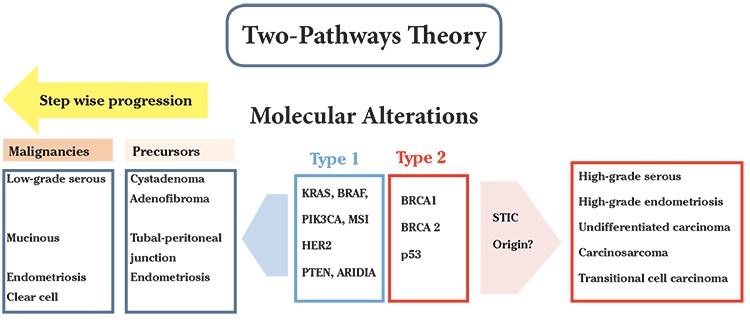
The two-pathways theory of ovarian cancer STIC: Serous tubal intraepithelial carcinoma

This theory was originally proposed by Kurman and Shih (3) in 2004, who sought to integrate the histological, clinical and genetic findings of ovarian cancer. They divided ovarian cancer into 2 types, namely type I and type II. Type I ovarian cancer consists of low-grade serous, mucinous, endometrioid, clear cell, and transitional histology types. Meanwhile, type II ovarian cancer consists of high-grade serous, undifferentiated and carcinosarcoma histology types (Figure 1) (3).

Precursor lesions are thought to originate in the ovary in type I ovarian cancer. In this, ovarian cancer grows slowly, tends to be benign, usually affects only the ovary in the diagnosis, and is genetically stable (19). Ovarian tumors undergo a series of morphologic changes on an ongoing basis and become ovarian cancer after surpassing the intermediate (borderline) phase. The pathogenesis of type I ovarian cancer is through the traditional pathway: ovarian surface epithelial inclusion cysts that receive proliferation stimulation from the environment, eventually transforming them into cancer cells. The most common genetic changes in type I ovarian cancers are KRAS and BRAF mutations, both of which can activate the oncogenic pathway MAPK (3,6,7).

In contrast to type I ovarian cancer, precursor lesions of type II ovarian cancer are thought to originate from outside the ovary, one of which is from the fallopian tube. Type II ovarian cancers tend to grow more aggressively, are genetically unstable, and are usually diagnosed at a more advanced stage. The majority of type II ovarian cancers exhibit TP53 gene mutations (50-80%), also overexpression of HER2/neu (10-20%) and AKT (12-18%) genes. Nearly half of all type II ovarian cancers are associated with BRCA1/2 gene mutations. Type II cancer cell precursors may originate from the fallopian tube, where a combination of TP53 mutations and environmental stressors such as inflammatory cytokines and reactive oxygen species cause secretory epithelial cells in the fallopian tubes to undergo neoplastic changes. Researchers showed that TP53 mutations were associated with lower parity, thus, ovulation was still considered the risk factor of TP53 gene mutation (3,19). In general, this theory is considered more capable of explaining the pathogenesis of ovarian cancer than other theories. However, it still lacks an understanding of the cancer development of non-ovarian origin (19).

### Prophylactic Surgical Methods for Ovarian Cancer

Primary prevention of ovarian cancer was mostly achieved by modifying risk factors and protective factors for ovarian cancer, based on epidemiologic data. For example, the use of oral contraceptives for at least 5 years reduces the risk of ovarian cancer by 50%. The same goes for parity, which reduces the risk of ovarian cancer by 50% when compared with nulliparity. However, these modifications have not shown a significant impact on the incidence of ovarian cancer in general. In addition, advocating the long-term use of oral contraceptives may increase the risk of breast cancer and thromboembolism (6,8). Clinical signs and symptoms of ovarian cancer are often non-specific and appear in a more advanced stage. No screening method has been proven effective in reducing the incidence of ovarian cancer, including periodic gynecologic examination, ultrasound study, and serum marker measurement. Calculating the possibility of ovarian cancer using CA125 may be useful. However, a single measurement may not be of value, thus serial testing is needed. Unfortunately, the rise in CA125 is not only associated with ovarian cancer, and there is no consensus on a threshold value to prompt surgical intervention for patients (4,20).

Currently, prophylactic surgical methods (either salpingectomy or salpingo-oophorectomy), have been proposed as a more effective primary prevention (6). In the past, prophylactic surgery was only intended for women at high risk of ovarian cancer, such as those with BRCA1/2 gene mutation. However, many studies suggest that not all ovarian cancers are related to genetic factors, thus prophylactic surgery is also considered useful for women in the general population. The fallopian tube, which is currently considered the initial location of ovarian cancer, has led to a trend shift from salpingo-oophorectomy to salpingectomy. The number of adverse effects caused by oophorectomy for young women also supports this tendency (7,21).

To reduce the incidence of ovarian cancer, it is estimated that 100 prophylactic salpingectomies should be performed to prevent 1 case of ovarian cancer (21). However, to date, prophylactic salpingo-oophorectomy is still considered the most effective preventive measure, and is associated with low incidence of surgical complications (9). In 2010, OVCARE began a campaign to perform prophylactic surgery at the time of hysterectomy or female sterilization procedures. It was estimated that the approach would reduce the incidence of ovarian cancer by 20-40% in the next 20 years (8). 

Salpingectomy is a simple procedure and can be performed simultaneously with or without a hysterectomy, for example in sterilization procedures. However, salpingectomy may disrupt blood flow to the ovary, impairing ovarian function, which is certainly not desired by young patients (8,9,22). Salpingectomy does not cause significant surgical risk and adds only minimal time. This procedure can be implemented during hysterectomy for benign disease, tubal sterilization, and other abdominal or pelvic surgery that gives access to gynecologic organs. However, salpingectomy during tubal sterilization may not be as popular because it is more difficult when compared with other techniques (20). Salpingectomy should include the total resection of the fallopian tube from the most distal fimbriae to the proximal portion up to the utero-tubal junction, without severing the collateral vasculature from mesosalpinx (23). Care must be taken in performing salpingectomy to avoid potential vascular compromise to both ovaries. When carefully executed, there is no significant decrease in ovarian function indicated by serum anti-mullerian hormone and follicle-stimulating hormone measurements (24). Salpingectomy adds 16 minutes to the operating time with hysterectomy, and 10 minutes in a sterilization procedure (20). Prophylactic salpingectomy is clearly an improvement in the efforts to prevent ovarian cancer. Nevertheless, the fallopian tube theory may not be the only pathogenesis of ovarian cancer. Hence, prophylactic salpingectomy may not prevent all ovarian cancers (22).

### Prophylactic Surgical Methods in BRCA1/2 Genes Mutation

Women with BRCA1 and BRCA2 mutations have a higher risk of ovarian cancer at the age of 70 years at 39-46% and 10-27%, respectively. The Society of Gynecologic Oncology recommends genetic testing for individuals with a high tendency for familial cancer (a first-degree or several close relatives with an inherited predisposition, a close relative carrying known BRCA1 or BRCA2 mutations, and a close relative with male breast cancer) (25). It is important to identify women at high risk, including the presence of BRCA mutation in the family, early-onset breast cancer, ovarian cancer at any age, male breast cancer, and Ashkenazi Jewish ancestry (26). Women with a first-degree relative with ovarian cancer have a three to four-fold increased risk of developing ovarian cancer (27).

One study showed that 54% of women with ovarian cancer and BRCA1 mutation were diagnosed before the age of 50 years, unusually diagnosed before the age of 40 years, and rarely before 30 years. Bilateral salpingo-oophorectomy should be considered for women with BRCA mutation after the age of 40 years once childbearing is complete because the onset of the disease is mostly after 40 years of age (26,28). Bilateral salpingo-oophorectomy reduces the risk of ovarian cancer by 75-96% and breast cancer by 50% when undertaken before menopause. The risk of primary peritoneal cancer after prophylactic surgery is reported as 2-4% (28).

The best timing for salpingo-oophorectomy in high-risk women is still controversial. It is agreed that the procedure must be performed as soon as possible given the potential of ovarian cancer. On the other hand, it may increase the risk of systemic complications in young women. Salpingo-oophorectomy is more effective if undertaken before menopause, but will lead to premature menopause in reproductive-aged women (7,22). Some authors recommend prophylactic salpingo-oophorectomy at the age of 40 or when reproductive function preservation is not desired (15,21). Women with BRCA mutations should be offered prophylactic surgery when childbearing is complete. The timing of surgery should also consider the age at onset of cancer in family members (29). Oophorectomy is associated with a rapid decline in serum estrogen and androgens, leading to postmenopausal symptoms and increased risk of various health problems (30). Prophylactic salpingo-oophorectomy is not without risk, however. Bilateral oophorectomy increased the risk of mortality associated with cardiovascular disease. In addition, this action can also increase the risk of parkinsonism, dementia, and osteoporosis (21,22,31). Studies reported that the risk of mortality due to cardiovascular disease was increased when performed before the age of 45-47.5 years (28,32). Premenopausal oophorectomy increases the risk of osteopenia and osteoporosis, and also causes a 20% decrease of trabecular bone, 18 months after surgery. The procedure is also associated with an increased risk of osteoporosis when performed before the age of 45 years. Thus, baseline bone density and follow-up every 1-2 years are recommended. The risk of cognitive impairment is greatest when oophorectomy is undertaken before the age of 49 years. Overall all-cause mortality was higher in women who underwent oophorectomy before the age of 45 years (33). Counseling should include the risk of death from ovarian cancer and the potential medical morbidities related to premature menopause (34).

Short-term sexual function seems to be less affected by oophorectomy, although studies are limited. In more than 50% of women, menopause-specific quality of life and sexual satisfaction were lower at 5 years after surgery (28). However, impairment of quality of life and sexual function in women who undergo bilateral salpingo-oophorectomy recover to baseline by 6 and 12 months (35). These adverse effects can be reduced by using hormone replacement therapy to some extent, but long-term use may decrease the benefit of oophorectomy on the breast as cancer prevention. Controversies regarding the risk-benefit comparison of oophorectomy exist. From the epidemiologic point of view, ovarian cancer is a far less common cause of female death (14,800 deaths/year) when compared with coronary heart disease (350,000 deaths/year) and hip for the actual (66,000 deaths/year) in the United States of America (USA). Also, around 10% of dementia in women is associated with a history of bilateral oophorectomy. These data conflict with the benefit of performing bilateral oophorectomy because preventing these problems (commonly associated with oophorectomy) seems much more important than preventing ovarian cancer due to their higher incidence. Patient’s age and family history are strong determinants for suggesting oophorectomy. Women with a known genetic predisposition should be recommended salpingo-oophorectomy after childbearing age (30).

Salpingectomy may be an option to avoid the adverse effects of oophorectomy. Histopathologic analysis of adnexa resected from BRCA-positive women revealed 4-17% had a cancerous lesion; 57-100% of cases were found in the distal portion of the fallopian tubes, characterized by an increase in the nuclear-cytoplasmic ratio, loss of nuclear polarity, nuclear pleomorphism, and loss of ciliated cells. This pathology is termed serous TIC (STIC). This lesion is found in almost 60% of patients with ovarian cancer, which indicates that the majority of cases are of tubal origin. It is estimated that 80-90% of BRCA-related ovarian cancers originate from the fallopian tube. Thus, only performing salpingectomy in BRCA-positive women may reduce the likelihood of having ovarian cancer as much as salpingo-oophorectomy, with the lowest risk of long-term complications. For young patients who wish to undergo prophylactic surgery, salpingectomy alone may provide more time to conceive via in vitro fertilization (36). Complete salpingectomy is preferred compared with fimbriectomy, although most BRCA-associated tubal lesions were found in the distal portion of the fallopian tube. The risk of ovarian cancer after hysterectomy with salpingectomy is 0.1-0.75% and the benefits of ovarian preservation decrease significantly after the age of 65 years (23). The risk of having repeat surgery for gynecologic problems after salpingectomy (with or without hysterectomy) is 0.89-5.5%, and the risk of developing ovarian cancer after hysterectomy with salpingectomy is reported as 0.1-0.75%. Therefore, salpingectomy alone or delayed oophorectomy can be a considerable choice for young patients (30). However, the effectiveness of salpingectomy alone is yet to be proven, and the benefit as a breast cancer prevention cannot be achieved (Finch, 2009). Considering the possibility of the ovarian origin of ovarian cancer, oophorectomy may still benefit women (7,21,22). 

The timing for prophylactic salpingectomy remains controversial. One may suggest that surgical prevention may be of more benefit if conducted at an earlier age. Some authors propose that salpingectomy should be performed after the age of 35 years in high-risk women (37). Unfortunately, currently, there is no large prospective study assessing the relationship between age-related risk reductions among women undergoing prophylactic surgery. 

Thus, for women with BRCA1/2 gene mutations, there are three options of prophylactic surgical procedures: (1) bilateral salpingo-oophorectomy, (2) salpingectomy alone, and (3) salpingectomy with delayed oophorectomy. A Markov Monte Carlo risk simulation study aimed at assessing the advantages of these options found that prophylactic salpingoopherectomy was the most effective strategy for the prevention of ovarian cancer. There are no data regarding the impact of two-staged surgery on quality of life, the percentage of women who decline the second surgery or delay the procedure long after natural menopause, and the overall impact on ovarian cancer incidence in this population (37). However, salpingectomy with delayed oophorectomy showed the best quality of life (21,22).

### Prophylactic Surgical Methods in General Population

In the general population, the lifetime risk of ovarian cancer is estimated to be around 1.4%. To date, there have been no recommendations for prophylactic surgical methods in the low-risk general population. For the low-risk population, oophorectomy is rarely recommended before the age of 40 years and highly recommended for women aged over 55 years (30). Prophylactic surgery has been implemented in several gynecologic procedures, such as sterilization and hysterectomy. Hysterectomy is the most common gynecologic procedure. In the USA, it is estimated that 600,000 hysterectomies are performed each year, and 55% are accompanied by bilateral salpingoopherectomy (7). Women who undergo hysterectomy without accompanying salpingectomy are at 7.8% higher risk of developing a disorder that ultimately requires salpingectomy, such as hydrosalpinges, infection, benign tumors, and ovarian cancer (8,21,38). When salpingectomy was integrated in the  hysterectomy procedure aimed for benign gynecological cases, it caused an increase in the number of salpingectomies 20 times in Canada. In addition, the method of female sterilization by salpingectomy is also recommended due to its protective effect against ovarian cancer compared with tubal ligation alone (22,39,40). For women in the general population who are undergoing hysterectomy, sterilization or pelvic and abdominal surgery, the decision to include ovarian cancer prevention should be made after detailed informed consent, including the risk and benefit of each procedure. Careful history taking, risk factor assessment, systemic and gynecologic disease evaluation should also be made. Low-risk women with certain gynecologic conditions (severe endometriosis, chronic pelvic inflammatory disease, ovarian neoplasm, and chronic pelvic pain) or medical conditions that may complicate repeat surgery (cardiopulmonary and hepato-renal disease, immunosuppression and morbid obesity) should consider prophylactic surgery. The rate of repeat surgery for various gynecologic indications ranges between 2.5% and 7.6% (29). In women aged 40 years and over, implementation of prophylactic surgery during hysterectomy and general surgery that permit access to the gynecologic organ may prevent ovarian cases by 5.2% and 10.9%, respectively. However, the decision of gynecologic or abdominal surgery should not be affected by the intention for salpingectomy (30).

Technically, the addition of a salpingectomy during hysterectomy does not increase the risk of complications and only slightly increases the duration of surgery (31). Salpingectomy performed during hysterectomy only increases the duration by about 16 minutes, and salpingectomy for female sterilization only increases the duration of surgery by 10 minutes compared with other procedures. No increased risk for blood transfusion need, prolonged hospital care, and postoperative re-admission have been reported (8,21,41).

Understanding the benefits of performing salpingectomy would encourage physicians to provide sufficient information regarding the procedure and may facilitate the patient’s decision making (22). In a Canadian survey involving obstetrics and gynecology specialists found that majority of physicians (68%) had been well-educated on the benefits of prophylactic salpingectomy and had or would add the procedure when performing a hysterectomy (38). Recent research showed that prophylactic salpingectomy procedures did not impair ovarian function. Morelli et al. found no significant differences in the levels of anti-mullerian hormone, follicle-stimulating hormone, the number of antral follicles, and the average diameter of the ovaries taken before surgery and 3 months after surgery (9,24).

The American College of Obstetrics and Gynecology issued its opinion regarding prophylactic salpingectomy as a preventive measure of ovarian cancer as follows (40):

 1. The surgeon and patient should discuss the potential benefits of the removal of the fallopian tubes during a hysterectomy in women at population risk of ovarian cancer who are not having an oophorectomy. 

2. When counseling women about laparoscopic sterilization methods, physicians can communicate that bilateral salpingectomy can be considered a method that provides effective contraception. 

3. Prophylactic salpingectomy may offer physicians the opportunity to prevent ovarian cancer in their patients.

4. Randomized controlled trials are needed to support the validity of this approach to reduce the incidence of ovarian cancer.

Kwon et al. (23) conducted a study using Markov Monte Carlo simulation models to assess the economic impact of prophylactic surgery during hysterectomy and sterilization in the general population. They found that hysterectomy with salpingectomy was less costly than hysterectomy alone or hysterectomy with bilateral salpingo-oophorectomy. However, hysterectomy with bilateral salpingo-oophorectomy was more effective in preventing ovarian cancer. They also found that even though salpingectomy for sterilization was more costly than tubal ligation, it was more effective at preventing ovarian cancer (23). Despite all the evidence that supports the role of prophylactic salpingectomy in preventing ovarian cancer, it has not yet become a guideline for the ovarian cancer prevention. Large-scale research is still required in the future (8,21).

Ovarian cancer is a heterogeneous disease, which consists of various histologic characteristics, with unclearly described pathogenesis. The fallopian tubes are thought to be the main location of the precursor lesions of most ovarian cancers, thus, prophylactic efforts are now directed towards surgical procedures for both the tubes and/or ovaries. In high-risk populations with BRCA1/2 gene mutations, salpingo-oophorectomy shows better effectiveness and is recommended for women aged over 40 years or when childbearing is complete. In young women, salpingectomy can be performed either alone or combined with late oophorectomy near the onset of natural menopause. In the low-risk general population, prophylactic salpingectomy still lacks a solid basis, but it may be offered during gynecologic procedures such as hysterectomy and female sterilization, or various pelvic and abdominal surgeries that allow access to the gynecologic organ.

Further research to validate the role of prophylactic surgery for ovarian cancer must be conducted, involving a larger and more diverse population. However, given the possible protective effects, the authors recommend that the available information should be delivered such that patients can choose whether to undergo prophylactic surgical procedures.
